# Supplementary material for: Structural Analysis and Antioxidant and Immunoregulatory Activities of an Exopolysaccharide Isolated from Bifidobacterium longum subsp. longum XZ01
Source: Molecules. 2023 Nov 6;28(21):7448. doi: 10.3390/molecules28217448 (PMC10649592; doi:10.3390/molecules28217448)
Supplement: Supplementary file 1 [file molecules-28-07448-s001.zip › molecules-2511878-supplementary.pdf]

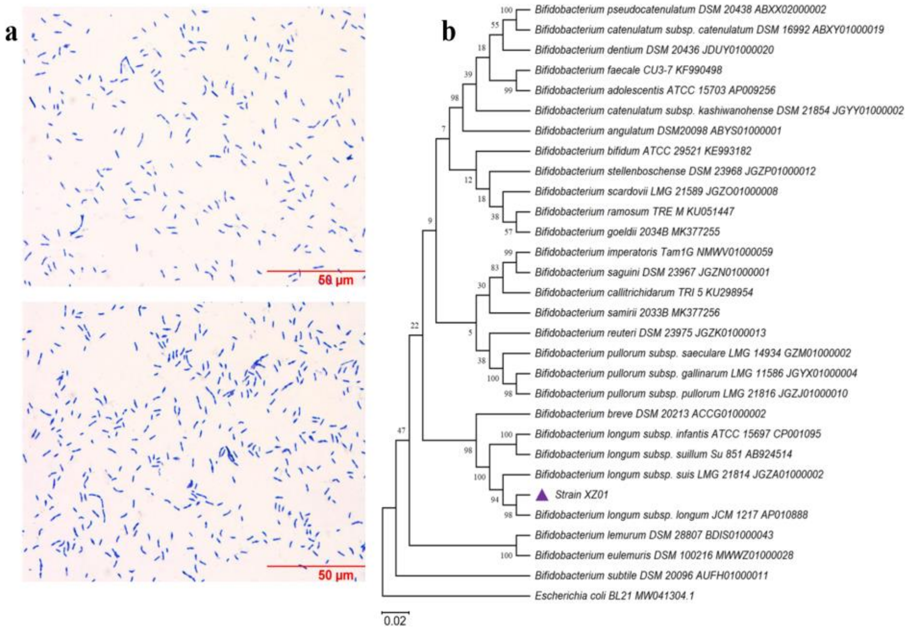

Figure S1. Gram staining results (**a**) and phylogenetic tree (**b**) of *Bifidobacterium longum* subsp. *longum* XZ01.

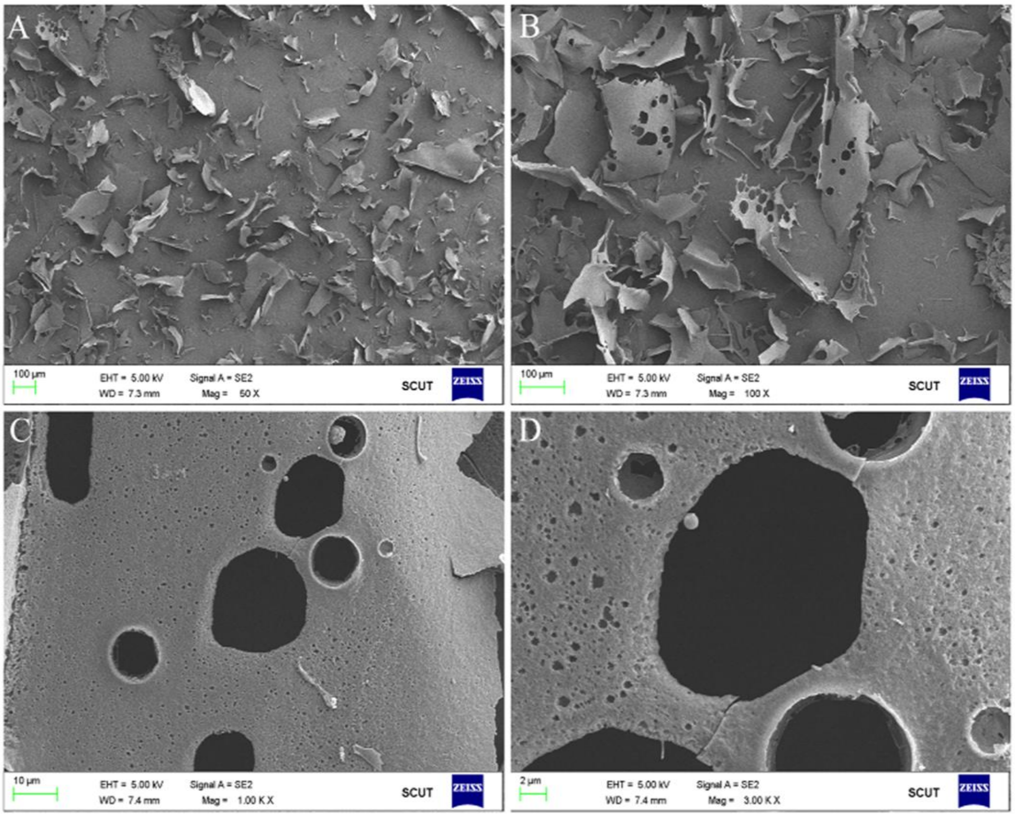

Figure S2. SEM images of S-EPS-1. A. 50 ×; B. 100 ×; C. 1000 ×; D. 3000 ×.

Table S1. PCR primer sequences.

Primer name		Sequence (5’→3’)
GAPDH	Forward	GTGTTCCTACCCCCAATGTGT
	Reverse	ATTGTCATACCAGGAAATGAGCTT
IL-1β	Forward	CTTCAGGCAGGCAGTATCACTC
	Reverse	TGCAGTTGTCTAATGGGAACGT
IL-6	Forward	CCTACCCCAATTTCCAATGCTC
	Reverse	GGTCTTGGTCCTTAGCCACTC
TNF-α	Forward	GATCGGTCCCCAAAGGGATG
	Reverse	GTGGTTTGTGAGTGTGAGGGT
